# (*E*)-3-(4-Bromo­phen­yl)-3-[3-(4-bromo­phen­yl)-1*H*-pyrazol-1-yl]prop-2-enal

**DOI:** 10.1107/S1600536809054749

**Published:** 2010-01-09

**Authors:** P. Ramesh, Ramaiyan Manikannan, S. Muthusubramanian, K. Ravichandran, M. N. Ponnuswamy

**Affiliations:** a Centre of Advanced Study in Crystallography and Biophysics, University of Madras, Guindy Campus, Chennai 600 025, India; b Department of Organic Chemistry, School of Chemistry, Madurai Kamaraj University, Madurai 625 021, India

## Abstract

There are two crystallographically independent mol­ecules in the asymmetric unit of the title compound, C_18_H_12_Br_2_N_2_O. In each mol­ecule, one of the bromo­phenyl rings lies almost in the plane of pyrazole unit [dihedral angles of 5.8 (3)° in the first mol­ecule and and 5.1 (3)° in the second] while the other ring is approximately perpendicular to it [dihedral angles of 80.3 (3) and 76.5 (3)°]. The crystal packing shows inter­molecular C—H⋯O inter­actions. The crystal studied was a racemic twin.

## Related literature

For the pharmacological and medicinal properties of pyrazole derivatives, see: Baraldi *et al.* (1998 ▶); Bruno *et al.* (1990 ▶); Cottineau *et al.* (2002 ▶); Londershausen (1996 ▶); Chen & Li (1998 ▶); Mishra *et al.* (1998 ▶); Smith *et al.* (2001 ▶). For a related structure, see: Jin *et al.* (2004 ▶). For hydrogen-bond motifs, see: Bernstein *et al.* (1995 ▶).
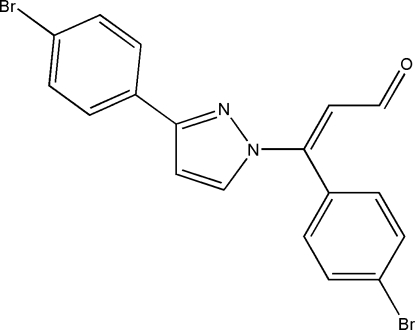


## Experimental

### 

#### Crystal data

C_18_H_12_Br_2_N_2_O
*M* *_r_* = 432.12
Orthorhombic, 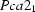

*a* = 9.2600 (3) Å
*b* = 9.3782 (3) Å
*c* = 37.9965 (4) Å
*V* = 3299.70 (15) Å^3^
*Z* = 8
Mo *K*α radiation
μ = 4.92 mm^−1^
*T* = 293 K
0.30 × 0.20 × 0.16 mm

#### Data collection

Bruker Kappa APEXII diffractometer
Absorption correction: multi-scan (*SADABS*; Sheldrick, 2001 ▶) *T*_min_ = 0.319, *T*_max_ = 0.455
17654 measured reflections
5353 independent reflections
3562 reflections with *I* > 2σ(*I*)
*R*_int_ = 0.032

#### Refinement

*R*[*F* ^2^ > 2σ(*F* ^2^)] = 0.037
*wR*(*F* ^2^) = 0.086
*S* = 1.01
5353 reflections
416 parameters
2 restraints
H-atom parameters constrained
Δρ_max_ = 0.94 e Å^−3^
Δρ_min_ = −0.68 e Å^−3^
Absolute structure: Flack (1983 ▶), 1831 Friedel pairs
Flack parameter: 0.226 (12)

### 

Data collection: *APEX2* (Bruker, 2004 ▶); cell refinement: *SAINT* (Bruker, 2004 ▶); data reduction: *SAINT*; program(s) used to solve structure: *SHELXS97* (Sheldrick, 2008 ▶); program(s) used to refine structure: *SHELXL97* (Sheldrick, 2008 ▶); molecular graphics: *ORTEP-3* (Farrugia, (1997 ▶)); software used to prepare material for publication: *SHELXL97* and *PLATON* (Spek, 2009 ▶).

## Supplementary Material

Crystal structure: contains datablocks global, I. DOI: 10.1107/S1600536809054749/bt5143sup1.cif

Structure factors: contains datablocks I. DOI: 10.1107/S1600536809054749/bt5143Isup2.hkl

Additional supplementary materials:  crystallographic information; 3D view; checkCIF report

## Figures and Tables

**Table 1:** Hydrogen-bond geometry (Å, °)

*D*—H⋯*A*	*D*—H	H⋯*A*	*D*⋯*A*	*D*—H⋯*A*
C8*B*—H8*B*⋯O1*B*^i^	0.93	2.50	3.419 (8)	172

Symmetry code: (i) 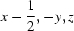.

**Fig. 1.**
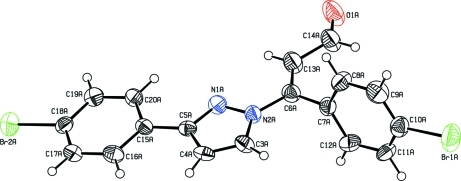
Perspective view of one of the two molecules in the asymmetric unit with the atomic numbering and 50% probability displacement ellipsoids.

**Fig. 2.**
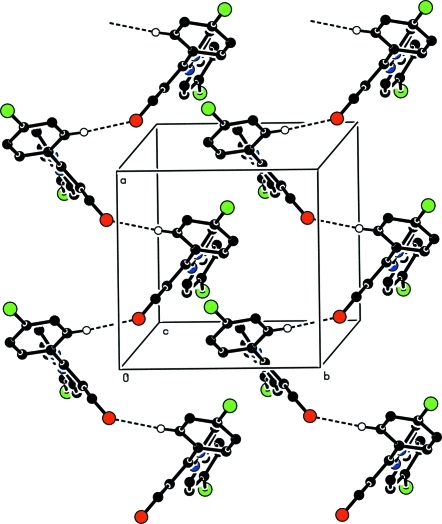
The crystal packing of the molecules viewed down c–axis. H atoms not involved in hydrogen bonding have been omitted for clarity.

## supplementary crystallographic information

### Comment

Pyrazole derivatives possess significant antiarrhythmic and sedative (Bruno *et al.*,  1990), hypoglycemic (Cottineau *et al.*, 2002), antiviral (Baraldi *et al.*,  1998), and pesticidal (Londershausen *et al.*, 1996) properties. Some pyrazole derivatives are successfully tested for their antifungal (Chen & Li, 1998), antihistaminic (Mishra *et al.*, 1998) and anti-inflammatory (Smith *et al.*,  2001) activities.  The crystallographic study of the title compound has been carried out to establish the molecular structure.

An ORTEP plot of the molecule is shown in Fig. 1. There are two crystallographically independent molecules in the asymmetric unit. One of the bromophenyl rings lies almost in the plane of the pyrazole moiety and the other ring is approximately perpendicular to it [dihidral angles [5.8 (3)° for C15A—C20A ring and 5.1 (3)° for C15B—C20B ring; 80.3 (3)° for C7A—C12A ring and 76.5 (3)° for C7B—C12B ring]. The vinyl aldehyde groups adopt extended conformation [C6A—C13A—C14A—O1A = -177.9 (7)° for molecule A and 179.4 (7)° for molecule B]. The sum of the bond angles at atoms N2A (359.9°) and N2B (360.0°) of the pyrazole ring in both molecules are in accordance with *sp*^2^ hybridization.

The molecular conformation is stabilized by weak intra molecular C—H···N interactions. The crystal packing shows intermolecular C—H···O interactions. Atom C8B at (x, y, z) donates a proton to atom O1B at (x - 1/2, -y, z), forming a C7 (Bernstein, 1995) zigzag chain running along the a axis as shown in Fig. 2

### Experimental

To a mixture of 1-(4-bromophenyl)-1-ethanone *N*-[(*E*)-1-(4-bromophenyl)ethylidene] hydrazone (0.003 mole) and 3 ml of dimethyl formamide kept in an ice bath at 0°C, phosphorus oxycholride (0.024 mole) was added dropwise for 5–10 minutes. The reaction mixture was then kept in a microwave oven at 600 W for 30–60 sec. The process of the reaction was monitored by TLC. After completion of the reaction, the reaction mixture was poured into crushed ice and extracted with dichloromethane. The organic layer was dried with anhydrous sodium sulfate. The different compounds present in the mixture were separated by column chromatography using petroleum ether and ethyl acetate mixture as eluent. This isolated compound was rectystalized in dichloromethane.

### Refinement

All H atoms were positioned geometrically (C—H = 0.93 Å) and allowed to ride on their parent atoms, with *U*_iso_(H) = 1.2*U*_eq_(C) for all H atoms.

### Crystal data

**Table:**

C_18_H_12_Br_2_N_2_O	*F*(000) = 1696
*M**_r_* = 432.12	*D*_x_ = 1.740 Mg m^−^^3^
Orthorhombic, *P**c**a*2_1_	Mo *K*α radiation, λ = 0.71073 Å
Hall symbol: P 2c -2ac	Cell parameters from 2356 reflections
*a* = 9.2600 (3) Å	θ = 2.1–26.7°
*b* = 9.3782 (3) Å	µ = 4.92 mm^−^^1^
*c* = 37.9965 (4) Å	*T* = 293 K
*V* = 3299.70 (15) Å^3^	Block, colorless
*Z* = 8	0.30 × 0.20 × 0.16 mm

### Data collection

**Table:**

Bruker Kappa APEXII diffractometer	5353 independent reflections
Radiation source: fine-focus sealed tube	3562 reflections with *I* > 2σ(*I*)
graphite	*R*_int_ = 0.032
ω and φ scans	θ_max_ = 26.7°, θ_min_ = 2.1°
Absorption correction: multi-scan (*SADABS*; Sheldrick, 2001)	*h* = −9→11
*T*_min_ = 0.319, *T*_max_ = 0.455	*k* = −11→8
17654 measured reflections	*l* = −47→28

### Refinement

**Table:**

Refinement on *F*^2^	Secondary atom site location: difference Fourier map
Least-squares matrix: full	Hydrogen site location: inferred from neighbouring sites
*R*[*F*^2^ > 2σ(*F*^2^)] = 0.037	H-atom parameters constrained
*wR*(*F*^2^) = 0.086	*w* = 1/[σ^2^(*F*_o_^2^) + (0.0332*P*)^2^ + 2.8025*P*] where *P* = (*F*_o_^2^ + 2*F*_c_^2^)/3
*S* = 1.01	(Δ/σ)_max_ = 0.002
5353 reflections	Δρ_max_ = 0.94 e Å^−^^3^
416 parameters	Δρ_min_ = −0.68 e Å^−^^3^
2 restraints	Absolute structure: Flack (1983), 1831 Friedel pairs
Primary atom site location: structure-invariant direct methods	Flack parameter: 0.226 (12)

### Special details

**Table:**

Geometry. All e.s.d.'s (except the e.s.d. in the dihedral angle between two l.s. planes) are estimated using the full covariance matrix. The cell e.s.d.'s are taken into account individually in the estimation of e.s.d.'s in distances, angles and torsion angles; correlations between e.s.d.'s in cell parameters are only used when they are defined by crystal symmetry. An approximate (isotropic) treatment of cell e.s.d.'s is used for estimating e.s.d.'s involving l.s. planes.
Refinement. Refinement of *F*^2^ against ALL reflections. The weighted *R*-factor *wR* and goodness of fit *S* are based on *F*^2^, conventional *R*-factors *R* are based on *F*, with *F* set to zero for negative *F*^2^. The threshold expression of *F*^2^ > σ(*F*^2^) is used only for calculating *R*-factors(gt) *etc*. and is not relevant to the choice of reflections for refinement. *R*-factors based on *F*^2^ are statistically about twice as large as those based on *F*, and *R*- factors based on ALL data will be even larger.

### Fractional atomic coordinates and isotropic or equivalent isotropic displacement parameters (Å^2^)

**Table:**

	*x*	*y*	*z*	*U*_iso_*/*U*_eq_	
Br1A	1.06404 (10)	0.03442 (11)	1.04136 (2)	0.0845 (3)	
Br1B	−0.30627 (8)	0.54380 (9)	0.52040 (2)	0.0701 (2)	
Br2A	0.53252 (8)	0.16474 (8)	0.66987 (2)	0.0689 (2)	
Br2B	0.20118 (8)	0.34086 (8)	0.888391 (19)	0.0687 (2)	
O1A	0.4407 (6)	0.3862 (6)	0.94905 (14)	0.0893 (18)	
O1B	0.2716 (6)	0.0770 (5)	0.61274 (14)	0.0782 (15)	
N1A	0.6924 (5)	0.1737 (5)	0.84642 (15)	0.0441 (13)	
N1B	0.0394 (5)	0.3223 (5)	0.71192 (13)	0.0386 (12)	
N2A	0.7688 (5)	0.1414 (5)	0.87580 (13)	0.0394 (12)	
N2B	−0.0373 (5)	0.3565 (5)	0.68274 (13)	0.0401 (12)	
C3A	0.8905 (7)	0.0679 (6)	0.86725 (18)	0.0456 (16)	
H3A	0.9595	0.0339	0.8829	0.055*	
C3B	−0.1573 (6)	0.4349 (6)	0.69043 (19)	0.0427 (16)	
H3B	−0.2241	0.4709	0.6745	0.051*	
C4A	0.8933 (7)	0.0536 (6)	0.83279 (19)	0.0451 (16)	
H4A	0.9637	0.0077	0.8195	0.054*	
C4B	−0.1601 (6)	0.4502 (6)	0.72623 (17)	0.0409 (15)	
H4B	−0.2292	0.4972	0.7397	0.049*	
C5A	0.7689 (6)	0.1212 (6)	0.82008 (16)	0.0357 (14)	
C5B	−0.0348 (7)	0.3788 (6)	0.73838 (16)	0.0358 (14)	
C6A	0.7229 (7)	0.1875 (6)	0.90911 (18)	0.0466 (17)	
C6B	0.0081 (6)	0.3060 (6)	0.64915 (17)	0.0390 (15)	
C7A	0.8119 (7)	0.1444 (7)	0.93918 (16)	0.0429 (16)	
C7B	−0.0733 (6)	0.3630 (7)	0.61901 (16)	0.0409 (16)	
C8A	0.9017 (7)	0.2424 (7)	0.95476 (18)	0.0520 (17)	
H8A	0.9111	0.3333	0.9452	0.062*	
C8B	−0.1625 (7)	0.2773 (7)	0.59906 (18)	0.0468 (16)	
H8B	−0.1745	0.1818	0.6050	0.056*	
C9A	0.9776 (8)	0.2055 (8)	0.9845 (2)	0.063 (2)	
H9A	1.0377	0.2725	0.9951	0.076*	
C9B	−0.2332 (7)	0.3330 (7)	0.57053 (18)	0.0527 (17)	
H9B	−0.2960	0.2759	0.5577	0.063*	
C10A	0.9667 (7)	0.0750 (8)	0.99855 (18)	0.0523 (17)	
C10B	−0.2125 (7)	0.4713 (7)	0.56087 (19)	0.0478 (17)	
C11A	0.8816 (9)	−0.0234 (8)	0.9832 (2)	0.059 (2)	
H11A	0.8749	−0.1140	0.9930	0.070*	
C11B	−0.1234 (7)	0.5587 (7)	0.58027 (18)	0.0486 (17)	
H11B	−0.1100	0.6535	0.5738	0.058*	
C12A	0.8043 (8)	0.0080 (7)	0.9532 (3)	0.058 (2)	
H12A	0.7477	−0.0615	0.9425	0.069*	
C12B	−0.0553 (8)	0.5044 (7)	0.6090 (2)	0.053 (2)	
H12B	0.0046	0.5631	0.6223	0.064*	
C13A	0.6043 (7)	0.2674 (7)	0.91238 (19)	0.0581 (18)	
H13A	0.5540	0.2924	0.8921	0.070*	
C13B	0.1155 (7)	0.2129 (6)	0.64759 (17)	0.0480 (16)	
H13	0.1556	0.1794	0.6685	0.058*	
C14A	0.5513 (8)	0.3163 (7)	0.9454 (2)	0.063 (2)	
H14A	0.6039	0.2936	0.9655	0.076*	
C14B	0.1728 (8)	0.1613 (7)	0.61425 (19)	0.0554 (18)	
H14	0.1320	0.1941	0.5934	0.067*	
C15A	0.7154 (6)	0.1352 (6)	0.78364 (17)	0.0375 (15)	
C15B	0.0164 (6)	0.3646 (6)	0.77424 (17)	0.0360 (14)	
C16A	0.7845 (7)	0.0686 (6)	0.75603 (19)	0.0472 (17)	
H16A	0.8673	0.0153	0.7604	0.057*	
C16B	−0.0520 (7)	0.4315 (6)	0.80212 (18)	0.0428 (16)	
H16B	−0.1355	0.4837	0.7978	0.051*	
C17A	0.7334 (7)	0.0793 (6)	0.72186 (18)	0.0464 (16)	
H17A	0.7822	0.0361	0.7033	0.056*	
C17B	−0.0001 (7)	0.4232 (7)	0.83575 (18)	0.0494 (17)	
H17B	−0.0479	0.4695	0.8540	0.059*	
C18A	0.6105 (7)	0.1544 (6)	0.71609 (16)	0.0420 (15)	
C18B	0.1237 (7)	0.3455 (6)	0.84255 (17)	0.0435 (15)	
C19A	0.5396 (6)	0.2233 (7)	0.74279 (19)	0.0456 (16)	
H19A	0.4566	0.2758	0.7381	0.055*	
C19B	0.1903 (6)	0.2769 (7)	0.81560 (18)	0.0470 (17)	
H19B	0.2726	0.2231	0.8201	0.056*	
C20A	0.5924 (6)	0.2141 (6)	0.77660 (17)	0.0413 (15)	
H20A	0.5451	0.2612	0.7948	0.050*	
C20B	0.1383 (7)	0.2854 (6)	0.78198 (18)	0.0398 (15)	
H20B	0.1858	0.2372	0.7640	0.048*	

### Atomic displacement parameters (Å^2^)

**Table:**

	*U*^11^	*U*^22^	*U*^33^	*U*^12^	*U*^13^	*U*^23^
Br1A	0.0906 (6)	0.1116 (6)	0.0512 (5)	0.0180 (5)	−0.0083 (5)	0.0153 (5)
Br1B	0.0723 (5)	0.0778 (5)	0.0601 (6)	0.0001 (4)	−0.0177 (4)	0.0176 (5)
Br2A	0.0836 (5)	0.0759 (5)	0.0474 (4)	0.0057 (4)	−0.0086 (4)	0.0055 (4)
Br2B	0.0748 (5)	0.0871 (5)	0.0442 (4)	0.0013 (5)	−0.0060 (4)	0.0059 (4)
O1A	0.096 (4)	0.116 (4)	0.056 (3)	0.053 (4)	−0.002 (3)	−0.022 (3)
O1B	0.082 (4)	0.077 (3)	0.075 (4)	0.032 (3)	0.016 (3)	−0.012 (3)
N1A	0.037 (3)	0.049 (3)	0.046 (4)	0.004 (3)	0.009 (3)	0.002 (3)
N1B	0.042 (3)	0.044 (3)	0.029 (3)	0.001 (2)	0.001 (2)	−0.001 (2)
N2A	0.040 (3)	0.044 (3)	0.035 (3)	0.003 (2)	0.003 (3)	−0.001 (2)
N2B	0.043 (3)	0.040 (3)	0.038 (3)	0.008 (3)	0.002 (3)	−0.006 (2)
C3A	0.046 (4)	0.044 (4)	0.047 (4)	0.001 (3)	−0.003 (3)	−0.012 (3)
C3B	0.033 (3)	0.036 (4)	0.059 (5)	0.009 (3)	−0.001 (3)	0.002 (3)
C4A	0.049 (4)	0.029 (3)	0.058 (5)	−0.001 (3)	0.003 (4)	−0.013 (4)
C4B	0.033 (3)	0.046 (4)	0.044 (4)	0.005 (3)	0.006 (3)	−0.002 (4)
C5A	0.033 (3)	0.030 (3)	0.044 (4)	−0.002 (3)	0.005 (3)	−0.001 (3)
C5B	0.045 (4)	0.025 (3)	0.038 (4)	−0.007 (3)	0.003 (3)	0.002 (3)
C6A	0.050 (4)	0.044 (4)	0.046 (5)	−0.003 (3)	0.007 (3)	−0.002 (3)
C6B	0.039 (3)	0.041 (4)	0.037 (4)	−0.006 (3)	−0.002 (3)	0.000 (3)
C7A	0.047 (4)	0.045 (4)	0.037 (4)	−0.004 (3)	0.002 (3)	0.002 (3)
C7B	0.045 (4)	0.041 (4)	0.037 (4)	0.004 (3)	0.004 (3)	−0.002 (3)
C8A	0.055 (4)	0.045 (4)	0.055 (4)	−0.004 (3)	−0.012 (4)	0.008 (3)
C8B	0.046 (4)	0.036 (3)	0.058 (4)	−0.009 (3)	−0.004 (3)	−0.002 (3)
C9A	0.057 (4)	0.067 (5)	0.066 (5)	−0.007 (4)	−0.007 (4)	−0.009 (4)
C9B	0.053 (4)	0.053 (4)	0.053 (4)	−0.004 (3)	−0.017 (4)	−0.009 (4)
C10A	0.050 (4)	0.059 (5)	0.048 (4)	0.005 (4)	0.004 (3)	0.006 (4)
C10B	0.046 (4)	0.047 (4)	0.050 (5)	0.002 (3)	−0.007 (3)	0.007 (3)
C11A	0.081 (5)	0.039 (4)	0.056 (5)	0.012 (4)	0.010 (4)	0.017 (4)
C11B	0.058 (4)	0.039 (4)	0.048 (4)	−0.005 (3)	0.000 (4)	0.003 (3)
C12A	0.074 (6)	0.034 (4)	0.064 (6)	−0.007 (3)	−0.001 (5)	0.002 (3)
C12B	0.059 (6)	0.054 (5)	0.046 (5)	−0.010 (3)	−0.013 (4)	−0.001 (3)
C13A	0.059 (5)	0.069 (4)	0.046 (4)	0.011 (4)	0.000 (3)	−0.008 (4)
C13B	0.056 (4)	0.049 (4)	0.039 (4)	0.009 (3)	−0.001 (3)	0.007 (3)
C14A	0.063 (5)	0.078 (5)	0.049 (4)	0.015 (4)	0.000 (3)	−0.012 (4)
C14B	0.063 (5)	0.056 (4)	0.048 (5)	0.010 (4)	0.006 (4)	−0.003 (4)
C15A	0.040 (4)	0.032 (3)	0.041 (4)	−0.008 (3)	0.010 (3)	−0.004 (3)
C15B	0.038 (3)	0.028 (3)	0.042 (4)	−0.003 (3)	0.007 (3)	0.001 (3)
C16A	0.037 (4)	0.046 (4)	0.058 (5)	0.006 (3)	0.003 (3)	−0.006 (4)
C16B	0.043 (4)	0.040 (4)	0.045 (4)	0.004 (3)	−0.002 (3)	−0.004 (4)
C17A	0.052 (4)	0.042 (4)	0.046 (4)	0.004 (3)	0.003 (3)	−0.006 (3)
C17B	0.050 (4)	0.058 (4)	0.040 (4)	0.005 (4)	0.009 (3)	−0.015 (3)
C18A	0.048 (4)	0.035 (3)	0.043 (4)	−0.001 (3)	0.000 (3)	0.007 (3)
C18B	0.045 (4)	0.045 (4)	0.040 (4)	−0.006 (3)	0.000 (3)	0.004 (3)
C19A	0.039 (4)	0.046 (4)	0.052 (5)	0.000 (3)	0.005 (3)	0.003 (3)
C19B	0.039 (4)	0.048 (4)	0.054 (5)	0.006 (3)	0.010 (3)	0.009 (3)
C20A	0.035 (3)	0.047 (4)	0.043 (4)	0.006 (3)	0.009 (3)	−0.001 (3)
C20B	0.046 (3)	0.036 (4)	0.038 (4)	0.002 (3)	0.007 (3)	−0.001 (3)

### Geometric parameters (Å, °)

**Table:**

Br1A—C10A	1.898 (7)	C8B—H8B	0.9300
Br1B—C10B	1.892 (7)	C9A—C10A	1.339 (9)
Br2A—C18A	1.901 (6)	C9A—H9A	0.9300
Br2B—C18B	1.884 (6)	C9B—C10B	1.361 (8)
O1A—C14A	1.224 (8)	C9B—H9B	0.9300
O1B—C14B	1.210 (8)	C10A—C11A	1.347 (10)
N1A—C5A	1.321 (8)	C10B—C11B	1.377 (9)
N1A—N2A	1.356 (7)	C11A—C12A	1.375 (12)
N1B—C5B	1.328 (7)	C11A—H11A	0.9300
N1B—N2B	1.355 (6)	C11B—C12B	1.361 (10)
N2A—N1A	1.356 (7)	C11B—H11B	0.9300
N2A—C3A	1.361 (8)	C12A—H12A	0.9300
N2A—C6A	1.403 (8)	C12B—H12B	0.9300
N2B—N1B	1.355 (6)	C13A—C14A	1.424 (10)
N2B—C3B	1.363 (7)	C13A—H13A	0.9300
N2B—C6B	1.425 (8)	C13B—C14B	1.456 (9)
C3A—C4A	1.316 (9)	C13B—H13	0.9300
C3A—H3A	0.9300	C14A—H14A	0.9300
C3B—C4B	1.368 (8)	C14B—H14	0.9300
C3B—H3B	0.9300	C15A—C16A	1.379 (8)
C4A—C5A	1.401 (9)	C15A—C20A	1.384 (8)
C4A—H4A	0.9300	C15B—C20B	1.382 (8)
C4B—C5B	1.416 (9)	C15B—C16B	1.384 (8)
C4B—H4B	0.9300	C16A—C17A	1.385 (9)
C5A—N1A	1.321 (8)	C16A—H16A	0.9300
C5A—C15A	1.477 (8)	C16B—C17B	1.367 (9)
C5B—N1B	1.328 (7)	C16B—H16B	0.9300
C5B—C15B	1.449 (8)	C17A—C18A	1.356 (8)
C6A—C13A	1.336 (8)	C17A—H17A	0.9300
C6A—C7A	1.465 (9)	C17B—C18B	1.383 (9)
C6B—C13B	1.325 (8)	C17B—H17B	0.9300
C6B—C7B	1.471 (8)	C18A—C19A	1.371 (9)
C7A—C8A	1.374 (9)	C18B—C19B	1.358 (9)
C7A—C12A	1.388 (9)	C19A—C20A	1.377 (9)
C7B—C8B	1.379 (8)	C19A—H19A	0.9300
C7B—C12B	1.389 (9)	C19B—C20B	1.367 (9)
C8A—C9A	1.375 (9)	C19B—H19B	0.9300
C8A—H8A	0.9300	C20A—H20A	0.9300
C8B—C9B	1.370 (9)	C20B—H20B	0.9300
			
C5A—N1A—N2A	105.1 (5)	C10A—C11A—C12A	121.1 (6)
C5B—N1B—N2B	104.7 (5)	C10A—C11A—H11A	119.4
N1A—N2A—C3A	110.4 (5)	C12A—C11A—H11A	119.4
N1A—N2A—C6A	121.0 (5)	C12B—C11B—C10B	119.0 (6)
C3A—N2A—C6A	128.5 (6)	C12B—C11B—H11B	120.5
N1B—N2B—C3B	112.3 (5)	C10B—C11B—H11B	120.5
N1B—N2B—C6B	120.0 (5)	C11A—C12A—C7A	119.3 (7)
C3B—N2B—C6B	127.7 (5)	C11A—C12A—H12A	120.4
C4A—C3A—N2A	107.8 (6)	C7A—C12A—H12A	120.4
C4A—C3A—H3A	126.1	C11B—C12B—C7B	121.4 (7)
N2A—C3A—H3A	126.1	C11B—C12B—H12B	119.3
N2B—C3B—C4B	106.6 (6)	C7B—C12B—H12B	119.3
N2B—C3B—H3B	126.7	C6A—C13A—C14A	123.1 (7)
C4B—C3B—H3B	126.7	C6A—C13A—H13A	118.5
C3A—C4A—C5A	106.3 (6)	C14A—C13A—H13A	118.5
C3A—C4A—H4A	126.8	C6B—C13B—C14B	122.1 (6)
C5A—C4A—H4A	126.8	C6B—C13B—H13	118.9
C3B—C4B—C5B	105.0 (6)	C14B—C13B—H13	118.9
C3B—C4B—H4B	127.5	O1A—C14A—C13A	124.0 (7)
C5B—C4B—H4B	127.5	O1A—C14A—H14A	118.0
N1A—C5A—C4A	110.4 (6)	C13A—C14A—H14A	118.0
N1A—C5A—C4A	110.4 (6)	O1B—C14B—C13B	122.3 (7)
N1A—C5A—C15A	119.8 (5)	O1B—C14B—H14	118.9
N1A—C5A—C15A	119.8 (5)	C13B—C14B—H14	118.9
C4A—C5A—C15A	129.8 (6)	C16A—C15A—C20A	118.5 (6)
N1B—C5B—C4B	111.4 (6)	C16A—C15A—C5A	121.2 (5)
N1B—C5B—C4B	111.4 (6)	C20A—C15A—C5A	120.3 (6)
N1B—C5B—C15B	120.4 (5)	C20B—C15B—C16B	117.0 (6)
N1B—C5B—C15B	120.4 (5)	C20B—C15B—C5B	121.1 (6)
C4B—C5B—C15B	128.2 (6)	C16B—C15B—C5B	121.9 (6)
C13A—C6A—N2A	120.4 (6)	C15A—C16A—C17A	121.4 (6)
C13A—C6A—C7A	123.0 (6)	C15A—C16A—H16A	119.3
N2A—C6A—C7A	116.6 (5)	C17A—C16A—H16A	119.3
C13B—C6B—N2B	118.7 (6)	C17B—C16B—C15B	121.9 (6)
C13B—C6B—C7B	126.1 (6)	C17B—C16B—H16B	119.1
N2B—C6B—C7B	115.2 (5)	C15B—C16B—H16B	119.1
C8A—C7A—C12A	118.8 (6)	C18A—C17A—C16A	118.4 (6)
C8A—C7A—C6A	119.4 (6)	C18A—C17A—H17A	120.8
C12A—C7A—C6A	121.7 (7)	C16A—C17A—H17A	120.8
C8B—C7B—C12B	118.5 (6)	C16B—C17B—C18B	119.7 (6)
C8B—C7B—C6B	121.5 (6)	C16B—C17B—H17B	120.1
C12B—C7B—C6B	119.8 (6)	C18B—C17B—H17B	120.1
C7A—C8A—C9A	119.7 (6)	C17A—C18A—C19A	121.8 (6)
C7A—C8A—H8A	120.2	C17A—C18A—Br2A	119.7 (5)
C9A—C8A—H8A	120.2	C19A—C18A—Br2A	118.5 (5)
C9B—C8B—C7B	120.0 (6)	C19B—C18B—C17B	119.0 (6)
C9B—C8B—H8B	120.0	C19B—C18B—Br2B	120.9 (5)
C7B—C8B—H8B	120.0	C17B—C18B—Br2B	120.0 (5)
C10A—C9A—C8A	121.3 (7)	C18A—C19A—C20A	119.4 (6)
C10A—C9A—H9A	119.4	C18A—C19A—H19A	120.3
C8A—C9A—H9A	119.4	C20A—C19A—H19A	120.3
C10B—C9B—C8B	120.6 (6)	C18B—C19B—C20B	121.1 (6)
C10B—C9B—H9B	119.7	C18B—C19B—H19B	119.4
C8B—C9B—H9B	119.7	C20B—C19B—H19B	119.4
C9A—C10A—C11A	119.8 (7)	C19A—C20A—C15A	120.4 (6)
C9A—C10A—Br1A	119.3 (6)	C19A—C20A—H20A	119.8
C11A—C10A—Br1A	120.8 (6)	C15A—C20A—H20A	119.8
C9B—C10B—C11B	120.5 (6)	C19B—C20B—C15B	121.2 (6)
C9B—C10B—Br1B	119.8 (5)	C19B—C20B—H20B	119.4
C11B—C10B—Br1B	119.7 (5)	C15B—C20B—H20B	119.4
			
C5A—N1A—N2A—C3A	−0.8 (6)	C8B—C9B—C10B—Br1B	177.8 (5)
C5A—N1A—N2A—C6A	176.8 (5)	C9A—C10A—C11A—C12A	−0.5 (12)
C5B—N1B—N2B—C3B	0.7 (6)	Br1A—C10A—C11A—C12A	176.3 (6)
C5B—N1B—N2B—C6B	−176.9 (5)	C9B—C10B—C11B—C12B	0.7 (10)
N1A—N2A—C3A—C4A	0.4 (7)	Br1B—C10B—C11B—C12B	−179.1 (6)
N1A—N2A—C3A—C4A	0.4 (7)	C10A—C11A—C12A—C7A	−1.5 (12)
C6A—N2A—C3A—C4A	−176.9 (6)	C8A—C7A—C12A—C11A	2.9 (11)
N1B—N2B—C3B—C4B	−1.1 (6)	C6A—C7A—C12A—C11A	−174.8 (7)
N1B—N2B—C3B—C4B	−1.1 (6)	C10B—C11B—C12B—C7B	0.3 (11)
C6B—N2B—C3B—C4B	176.3 (6)	C8B—C7B—C12B—C11B	0.0 (11)
N2A—C3A—C4A—C5A	0.2 (7)	C6B—C7B—C12B—C11B	176.8 (7)
N2B—C3B—C4B—C5B	0.9 (7)	N2A—C6A—C13A—C14A	179.3 (6)
N2A—N1A—C5A—C4A	0.9 (6)	C7A—C6A—C13A—C14A	−1.2 (10)
N2A—N1A—C5A—C15A	178.9 (5)	N2B—C6B—C13B—C14B	−177.0 (5)
C3A—C4A—C5A—N1A	−0.7 (7)	C7B—C6B—C13B—C14B	4.0 (10)
C3A—C4A—C5A—C15A	−178.5 (6)	C6A—C13A—C14A—O1A	−177.9 (7)
N2B—N1B—C5B—C4B	−0.1 (6)	C6B—C13B—C14B—O1B	179.4 (7)
N2B—N1B—C5B—C15B	−179.4 (5)	N1A—C5A—C15A—C16A	−173.0 (5)
C3B—C4B—C5B—N1B	−0.5 (7)	C4A—C5A—C15A—C16A	4.7 (9)
C3B—C4B—C5B—N1B	−0.5 (7)	N1A—C5A—C15A—C20A	5.9 (8)
C3B—C4B—C5B—C15B	178.7 (6)	C4A—C5A—C15A—C20A	−176.4 (6)
N1A—N2A—C6A—C13A	−1.6 (8)	N1B—C5B—C15B—C20B	−3.8 (8)
C3A—N2A—C6A—C13A	175.4 (6)	C4B—C5B—C15B—C20B	177.0 (6)
N1A—N2A—C6A—C7A	178.9 (5)	N1B—C5B—C15B—C16B	174.8 (5)
C3A—N2A—C6A—C7A	−4.1 (9)	C4B—C5B—C15B—C16B	−4.4 (9)
N1B—N2B—C6B—C13B	7.6 (8)	C20A—C15A—C16A—C17A	0.0 (8)
C3B—N2B—C6B—C13B	−169.6 (6)	C5A—C15A—C16A—C17A	178.9 (6)
N1B—N2B—C6B—C7B	−173.3 (5)	C20B—C15B—C16B—C17B	1.5 (9)
C3B—N2B—C6B—C7B	9.5 (8)	C5B—C15B—C16B—C17B	−177.1 (6)
C13A—C6A—C7A—C8A	−75.2 (8)	C15A—C16A—C17A—C18A	−1.7 (9)
N2A—C6A—C7A—C8A	104.3 (7)	C15B—C16B—C17B—C18B	−0.3 (10)
C13A—C6A—C7A—C12A	102.4 (9)	C16A—C17A—C18A—C19A	2.4 (9)
N2A—C6A—C7A—C12A	−78.1 (8)	C16A—C17A—C18A—Br2A	−177.3 (5)
C13B—C6B—C7B—C8B	67.2 (9)	C16B—C17B—C18B—C19B	−1.1 (9)
N2B—C6B—C7B—C8B	−111.8 (6)	C16B—C17B—C18B—Br2B	177.0 (5)
C13B—C6B—C7B—C12B	−109.5 (8)	C17A—C18A—C19A—C20A	−1.3 (9)
N2B—C6B—C7B—C12B	71.5 (8)	Br2A—C18A—C19A—C20A	178.4 (5)
C12A—C7A—C8A—C9A	−2.4 (10)	C17B—C18B—C19B—C20B	1.1 (9)
C6A—C7A—C8A—C9A	175.4 (6)	Br2B—C18B—C19B—C20B	−176.9 (5)
C12B—C7B—C8B—C9B	−1.3 (10)	C18A—C19A—C20A—C15A	−0.5 (9)
C6B—C7B—C8B—C9B	−178.0 (6)	C16A—C15A—C20A—C19A	1.1 (8)
C7A—C8A—C9A—C10A	0.5 (11)	C5A—C15A—C20A—C19A	−177.8 (5)
C7B—C8B—C9B—C10B	2.4 (10)	C18B—C19B—C20B—C15B	0.2 (9)
C8A—C9A—C10A—C11A	1.0 (11)	C16B—C15B—C20B—C19B	−1.5 (8)
C8A—C9A—C10A—Br1A	−175.8 (5)	C5B—C15B—C20B—C19B	177.2 (5)
C8B—C9B—C10B—C11B	−2.0 (10)		

### Hydrogen-bond geometry (Å, °)

**Table:**

*D*—H···*A*	*D*—H	H···*A*	*D*···*A*	*D*—H···*A*
C13A—H13A···N1A	0.93	2.43	2.779 (9)	102
C13B—H13···N1B	0.93	2.38	2.743 (8)	103
C8B—H8B···O1B^i^	0.93	2.50	3.419 (8)	172

Symmetry codes:  (i) *x*−1/2, −*y*, *z*.
